# Vaccinia Virus in Blood Samples of Humans, Domestic and Wild Mammals in Brazil

**DOI:** 10.3390/v10010042

**Published:** 2018-01-18

**Authors:** Marina G. Peres, Thais S. Bacchiega, Camila M. Appolinário, Acácia F. Vicente, Mateus de Souza Ribeiro Mioni, Bruna L. D. Ribeiro, Clóvis R. S. Fonseca, Vanessa C. Pelícia, Fernando Ferreira, Graziele P. Oliveira, Jonatas S. Abrahão, Jane Megid

**Affiliations:** 1 Faculdade de Medicina Veterinária e Zootecnia, UNESP—Universidade Estadual Paulista, Botucatu CEP 18618-970, Brazil; marinageavet@yahoo.com.br (M.G.P.); tatabacch@hotmail.com (T.S.B.); camilaapp.vet@gmail.com (C.M.A.); acaciavicente@hotmail.com (A.F.V.); mateusmioni@yahoo.com.br (M.d.S.R.M.); brunadevider@gmail.com (B.L.D.R.); crsfonseca2@yahoo.com.br (C.R.S.F.); vcpelicia@yahoo.com.br (V.C.P.); 2 Faculdade de Medicina Veterinária e Zootecnia, USP—Universidade de São Paulo, São Paulo CEP 05508-270, Brazil; fernando@vps.fmvz.usp.br; 3 Instituto de Ciências Biológicas, UFMG—Universidade Federal de Minas Gerais, Belo Horizonte CEP 31270-901, Brazil; graziufmg@yahoo.com.br (G.P.O.); jonatas.abrahao@gmail.com (J.S.A.)

**Keywords:** Vaccinia virus, blood samples, humans, domestic mammals, wild mammals, public health, epidemiology, transmission

## Abstract

Outbreaks of Vaccinia virus (VACV) affecting cattle and humans have been reported in Brazil in the last 15 years, but the origin of outbreaks remains unknown. Although VACV DNA have been already detected in mice (*Mus musculus*), opossums (*Didelphis albiventris*) and dogs during VACV zoonotic outbreaks, no transmission to cattle or humans from any of these were reported during Brazilian outbreaks. In this work, we assessed the PCR positivity to VACV in blood samples of cows and other domestic mammals, wild rodents and other wild mammals, and humans from areas with or without VACV infection reports. Our results show the detection of VACV DNA in blood samples of cows, horse and opossums, raising important questions about VACV spread.

## 1. Introduction

The first official report of a Vaccinia virus (VACV) outbreak in cattle and humans in Brazil was recorded in 1999 at Cantagalo city, Rio de Janeiro State [1]. Since then, outbreaks have been described in several regions of the country [2,3,4,5,6,7,8,9]. It was believed that VACV reemergence in zoonotic outbreaks was related to the VACV vaccines strain used during campaigns of World Health Organization (WHO) against smallpox, and their possible adaptation to some wild host [10]. Now it is known that VACV isolated in Brazilian outbreaks is divergent from VACV vaccines strains, but the origin of outbreaks remains unknown [11,12].

In this way, a model of transmission has been proposed in which peridomestic rodents act as a link between domestic animals and wildlife [13]. In fact, peridomestic rodents like *Mus musculus*, *Rattus rattus* and *Rattus norvergicus* act as host reservoirs of cowpox virus in Europe [14]. Then the members of Rodentia order have been targeted of research looking for explaining its possible role in the VACV spread [15,16,17,18].

Despite VACV has once isolated from peritoneum and testicles samples of *Mus musculus*, and VACV DNA was once detected in blood samples of opossums (*Didelphis albiventris*) and dogs, during VACV outbreaks respectively [13,19], no transmission to cows or humans for any of those species were established during the Brazilian VACV outbreaks.

In Brazil, during a VACV zoonotic outbreak, VACV DNA was detected in blood samples of a milker and in the blood of her two daughters that were not engaged in any exposure activity [20]. VACV DNA has been also detected in blood samples of smallpox vaccine recipients, as well as, in blood samples of experimentally infected cows and mice [17,21,22]. Accordingly, we accessed the PCR positivity for VACV in blood samples from humans, cows and other domestic and wild mammals from areas with or without official reports of VACV zoonotic outbreaks in the central west region of São Paulo State, Brazil.

## 2. Materials and Methods

All animals and humans samples in the present study were tested previously for the presence of antibodies anti-*Orthopoxvirus* (OPV) [18].

This study was approved by Ethical Committee of Animal Uses in Veterinary Medicine and Animal Production College of São Paulo State University “Júlio de Mesquita Filho”(number 114/2015-CEUA, 04/09/2015) and by the Ethical Committee in Research of Medicine College of the aforementioned university (CEP3605-2010).

**Figure 1:**
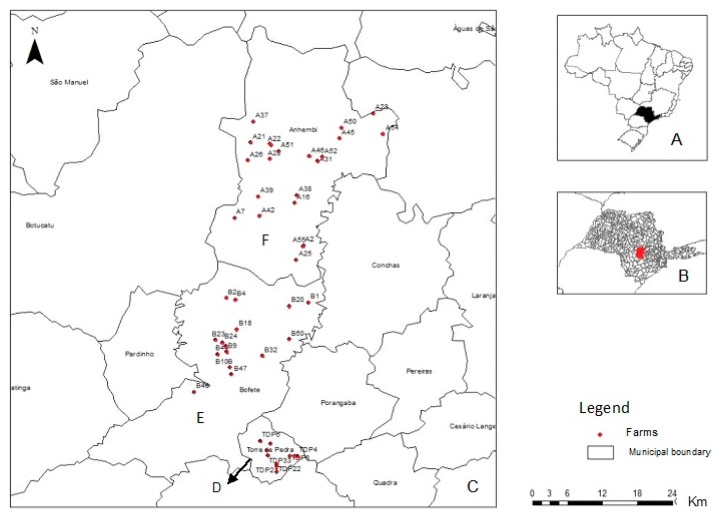
Map of sites sampling, (**A**) with São Paulo State in black. The São Paulo state map; (**B**) with Torre de Pedra, Bofete and Anhembi in red. The Map of São Paulo State; (**C**) showing the sites of sampling; the points in red correspond to farms in Torre de Pedra (**D**); Bofete (**E**) and Anhembi (**F**). Source: Peres et al., 2013 [18].

Samples were collected in three counties with and without official reports of VACV zoonotic outbreaks: Torre de Pedra (23°14′58.76″ S, 48°11′39.49″ W), where outbreaks were previously registered [3,6], Bofete (23°05′54.51″ S, 48°11′26.61″ W) and Anhembi (23°05′54.51″ S, 48°11′26.61″ W) in which histories of outbreaks are unknown (Figure 1).

The total numbers of farms included in the study was calculated based on the populations of the farms in the three counties, respectively, a 5% prevalence of positive farms (at least one positive sample), and a 5% margin of error using Epi Info 3.5.4 (CDC, Atlanta, GA, USA). Samples were selected randomly from 48 farms—11 in Torre de Pedra, 15 in Bofete, and 22 in Anhembi (Figure 1).

The minimum number of sampled cows was determined using the program HERDACC3.0^®^ for each farm, assuming a PCR sensitivity of 80%, a specificity of 99.9%, and a proportion of infected animals in a positive herd of 20%. The minimum number of lactating cows that needed to be sampled to ensure a minimum sensitivity and specificity of 95% was 20 animals. In a cattle herd with more than 20 animals, only 20 lactating cows selected randomly were sampled, but in a herd with fewer than 20 animals, samples were collected from all animals. From other species (horse, sheep, swine, dogs and cats), one to five samples were collected.

Blood samples were collected from February to April 2011, by mammary vein puncture or jugular puncture from cows and by jugular puncture from other domestic species, and stored at −20 °C until the PCR test. All animals were examined to presence of characteristic clinical signs, as pustules and crusts.

Capture of wild mammals was conducted from May to September 2011 and was authorized by the Brazilian Institute of Environment and Natural Resource Renewable (IBAMA), the Chico Mendes Biodiversity Conservation Institute (ICM-Bio) by Biodiversity Information and Authorization System (SISBIO) under authorization number 23918-1.

Tomahawk traps with chicken bait were used for capture of wild mammals and pitfall and Sherman traps with a bait consisting of a mixture of peanut cream, canned sardine, cornmeal and oatmeal were used for wild rodents capture. Five traps nights in each farm were required to capture animals in native forest areas surrounding them (consisting of a transitional Atlantic Forest and Cerrado).

Wild mammals were anesthetized with tiletamine and zolazepan (Zoletil^®^) using the recommended dose for each species [23] and blood samples were collected by jugular puncture. Due to infectious agents related to rodents, like *Hantavirus* spp, all wild rodents procedure before, during and after blood samples collection, were performed with uses of personal protective equipment (EPP), as previously described [18]. All collected blood samples were stored at −20 °C until the PCR test. All animals were examined to presence of characteristic clinical signs, as pustules and crusts.

Blood samples of farmers, rural workers and their families were collected by nurses by cephalic vein puncture during October and November 2011, and stored at −20 °C until the PCR test.

Viral DNA was extracted using the Invisorb^®^ Spin Blood Mini Kit (Stratec Molecular, Berlin, Germany). A nested PCR was used for the amplification of the Vaccinia growth factor (*vgf*) gene [24]. The nested PCR was carried out in a two-step reaction protocol. In the first step, were used the OPV primers (vgfF: CGCTGCTATGATAATCAGATCATT and vgfR: GATATGGTTGTGCCATAATTTTTAT). In the nested step, the pair of internal OPV primers (vgfF2: ACACGGTGACTGTATCCA and vgfR2: CTAATACAAGCATAATAC) were used [24]. In the first step, 2 μL of template were added to 18 μL of the PCR reaction mixture containing 0.4 mM of OPV primers (VGF-F and VGF-R), 10 mM dNTPs, 2.0 mM MgCl_2_, 500 ng Bovine Serum Albumin (BSA) and 2 U of Taq DNA polymerase, using the manufacturer’s supplied 10× buffer. Reactions were performed using the following protocol: incubation at 95 °C for 9 min; 30 cycles of denaturation (94 °C, 1 min), annealing (45 °C, 1 min) and extension (72 °C, 1 min); final extension (72 °C, 10 min). The nested PCR step was carried out using 1 μL of undiluted first PCR product as template. The same chemical and thermal conditions were used, but using internal OPV primers (vgfF2 and vgfR2). PCR sensitivity was determined with decimal serial dilutions ranging from 10^4^ to 1PFU of VACV-WR as templates and the sensitivity was defined by the highest viral dilution detected by PCR [24]. The *vgf* gene is a conserved *Orthopoxvirus* (OPV) gene, widely used as a PCR target, in diagnostic and phylogenetic of Brazilian VACV outbreaks [22,24,25]. The *vgf* PCR products were purified by uses of the Illustra^®^ GFX PCR and Gel Band Purification Kit (GE Healthcare Life Science, Freiburg, Germany) and then, were submitted to cloning using the pGEM^®^-T Easy Vector System I (Promega, Madison, WI, USA) at Virus Laboratory of Bioscience Institute of the Federal University of Minas Gerais—UFMG. Cloned samples were submitted to gene sequencing and obtained sequences aligned with previously published VACV sequences from GenBank using the Clustal W method, and the alignments were manually checked with MEGA version 7.0 software (Arizona State University, Phoenix, AZ, USA). A phylogenetic tree was constructed using the neighbor-joining method, the Tamura-3 model of nucleotide substitutions and a bootstrap of 1000 replicates.

## 3. Results

### 3.1. Missing Values

A total of 48 farms was randomly chosen for the study, however one farm from Torre de Pedra didn’t authorize the study, resulting in a total of 47 farms sampled. A number of 138 wild rodents were captured but collection of blood samples were successfully performed in 103 animals, then results are shown only for these animals.

### 3.2. Samples Collected and Positivity

A total of 1331 blood samples—148 from humans, 688 from cows, 44 from sheep, 22 from swine, 117 from horses, 114 from dogs, 7 from cats, 57 from white-eared opossums (*Didelphis albiventris*), 16 from black-eared opossums (*Didelphis aurita*), 6 from Brazilian gracile opossums (*Grcilinanus microtarsus*), 4 from crab-eating foxes (*Cerdocyon thous*), 4 from coatis (*Nasua nasua*), 1 from ocelot (*Leopardus pardalis*), and 103 from wild rodents (4 *Akodon montensis,* 4 *Nectomys squamipes*, 4 *Calomys tener*, 13 *Sooretamys agouya,* 17 *Oligoryzomys flavescens*, 61 *Oligoryzomys nigripes,*)—were collected (Table 1).

From collected samples, eight (0.6%) were positive for amplification of *vgf* gene in the PCR test. Positivity were observed in four cows (0.6% of sampled cows), one horse (0.8% of sampled horse), two black-eared opossums (12.5% of sampled *D. aurita*) and one white-eared opossum (1.7% of sampled *D. albiventris*) (Table 1). The positive animals were from Anhembi (0.7%) and Bofete (0.7%), and no positivity were detected in blood samples from Torre de Pedra (Table 2), where VACV zoonotic outbreaks were previously reported [3,6].

Of the total analyzed samples, one animal from Bofete was simultaneously positive for the detection of DNA in the blood and neutralizing antibodies (titer equal to 16) against OPV in the serum respectively, while the other seven PCR positive samples were from seronegative animals (Table 3), and serological results previously published [18]).

The PCR positive animals were from six different milking farms, four located in Anhembi county and two from Bofete county, both counties without official reports of VACV outbreaks. PCR positivity in two animals (a cow and a horse) from the same farm were observed in one milking farm from Anhembi and other milking farm from Bofete (two *D. aurita*). The farms that presented the PCR-positive animals are those that had previous seropositivity to OPV in animals and humans (Table 4).

### 3.3. Sequencing and Analysis of the Sequences

**Figure 2:**
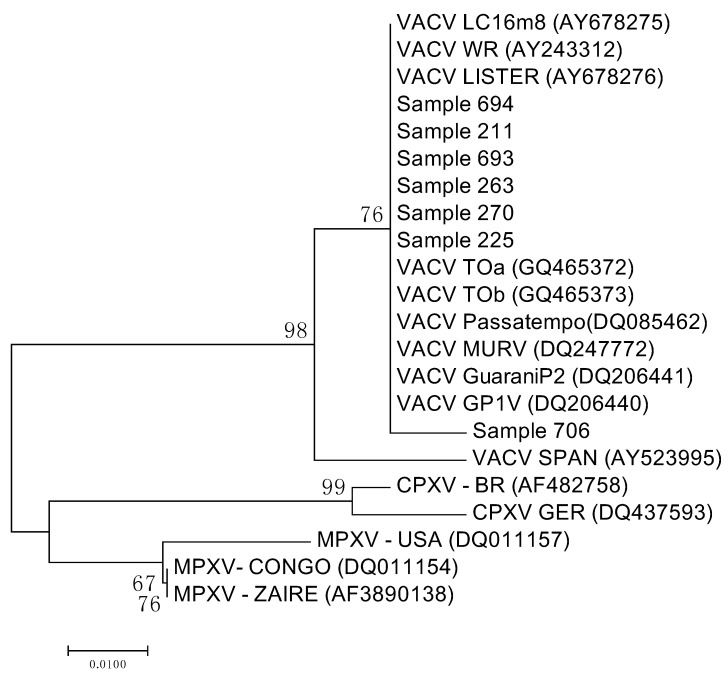
Phylogenetic tree based on the OPV nucleotide sequence of the *vgf* gene showing blood samples cluster (Sample 694, Sample 211, Sample 693, Sample 263, Sample 270, Sample 225, Sample 706). Sample 694 = blood sample of *Didelphis aurita;* Sample 211 = blood sample of cow; Sample 693 = blood sample of *Didelphis aurita;* Sample 263 = blood sample of cow; Sample 270 = blood sample of horse; Sample 225 = blood sample of cow; Sample 706 = blood sample of *Didelphis albiventris*.

Although all eight positive samples were sequenced just seven could be considered for the phylogenetic analysis due to low coverage of one of them. Thus, the sequencing and phylogenetic tree based on the seven orthopoxvirus nucleotide sequence of the *vgf* gene revealed that our strains (Sample 694, Sample 211, Sample 693, Sample 263, Sample 270, Sample 706) clustered with the Brazilian VACVs (i.e., TOa, TOb, Passatempo, MURV, GP1V, GP2V) and vaccine VACVs (i.e., Lc16m2, WR, and Lister), which characterized it as a Vaccinia virus (Figure 2). The percentage of identity between the different samples is presented in the Table S1.

## 4. Discussion

Vaccinia virus (VACV), as other *Orthopoxvirus* (OPV), exhibit a tropism for epithelial cells. During outbreaks, diagnoses are done preferably through the analysis of scabs [1,2,3,4,5,6,7]. In the present study, we assessed the presence of VACV in bovine herds and in other domestic and wild mammals, as well as in humans from milk farms located in areas with and without official reports of zoonotic outbreaks. Once the 47 milk farms were not in the proximity of a VACV outbreak, and considering that VACV DNA has been detected in blood samples of experimentally and naturally infected animals, even as in blood samples of naturally infected humans [15,17,19,20,22], we investigated the presence of VACV DNA in blood samples collected of healthy animals and humans from these properties.

Viral DNA could be detected in the blood samples of one horse, three opossums (one *Didelphis albiventris* and two *Didelphis aurita*) and four cows, all apparently healthy, from areas without official reports of VACV outbreaks (Anhembi and Bofete counties) in São Paulo State. To the best of our knowledge, this is the first time that VACV DNA is detected in blood samples of naturally infected cows, horse and *Didelphis aurita*.

All sampled animals and humans of the present study were previously tested for the presence of OPV neutralizing antibodies, and seropositivity was observed in humans, cows, horses, swine, dog, cat, opossums (*D. aurita*; *D. albiventris*), coati (*Nasua nasua*), and in wild rodents (*Oligoryzomys nigripes*, *Oligoryzomys flavescens*, *Sooretamys angouya*) [18]. In the present study, only one cow showed simultaneous detection of viral DNA and neutralizing antibodies, in blood and serum samples respectively, while the other three cows and the horse showed only the presence of viral DNA without detectable neutralizing antibodies. A similar situation was observed during a VACV zoonotic outbreak when serum samples of unaffected humans showed only viral DNA, or both, viral DNA and neutralizing antibodies [20]. The absence of clinical signs associated with the presence of viral DNA in the blood with or without simultaneous presence of neutralizing antibodies suggest a systemic and subclinical infection or an early stage of infection that could evolve to future clinical manifestation.

Apparently humans acquire clinical infection when in contact with viral particles disposed on bovine or human crusts [3,4,6,8], however subclinical human infections has been described with the detection of viral DNA in the blood, been related to other routes of infection, as fomites in the household or through the consume of raw milk and cheese [20]. In our study all human blood samples tested negative for the presence of viral DNA, however neutralizing antibodies were previously detected in 17% of them, and seropositivity was directly correlated with age, and higher in older persons [18]. Both possibilities were suggested, the smallpox vaccine memory or lifelong exposure of farmer to the circulating virus in domestic animals, especially cows [18], but considering that we had seropositive results in people that had never been vaccinated with smallpox vaccine [18], it is possible to suggest that the absence of viral DNA in the blood associated with neutralizing antibodies, may be due to constant contact with domestic animals clinically or sub-clinically infected once the seropositivity was observed in regions with and without VACV infection reported.

The opossums (*D. aurita*; *D. albiventris*) in which blood samples viral DNA were detected, were previously classified as seronegative. This classification was based on the amount of neutralizing antibodies presented by these animals that was insufficient to induce 50% or more of reduction of viral plaques [18]. However, the presence of viral DNA in the blood, associated with the low percentage (9% in *D. albiventris*; 13% and 14% respectively in both *D. aurita*) of reduction of viral plaques may suggest acute infection with initial production of neutralizing antibodies; these animals would present clinical signs in the future, but subclinical infection cannot be ruled out. In a previous report [19], *Didelphis albiventris*, apparently healthy, also showed the presence of viral DNA in their blood samples, during a VACV zoonotic outbreak and subclinical infection as well as their possible role as VACV reservoir was questioned.

Concerning the role of wild rodents in VACV spread, our results didn’t show viral DNA in blood of sampled wild rodents from this sampling area, and low percentage of seropositivity were previously detected in these animals [18]. However, VACV DNA was detected in the feces of *O. flavescens*, *O. nigripes* and *S. angouya*, as well as in urine of *O. flavescens* from the same area. The simultaneous detection of VACV DNA in feces and neutralizing antibodies in serum samples of two *O. flavescens* from Bofete were observed (unpublished work [18,26]). These animals were apparently healthy, and showed 54% and 59% of reduction in viral plaques being considered positives to OPV antibodies [18]. These finds together suggest acute infection with initial production of neutralizing antibodies in these animals that could lead them to develop or not clinical signs. In fact, sentinel mice exposed to feces of experimentally infected cows and mice didn’t show any clinical sign but viral DNA could be detected in their blood, organs and feces samples suggesting subclinical infection [15,17].

Taking our findings together, we cannot rule out the possibility of these animals (DNA positive) eventually developing clinical signs, but considering that no VACV-like outbreaks were reported in the three sampled counties, from the initial period of samples collection until now, these animals (DNA positive) presumably subclinically infected, can sustain the viral circulation in the sampling area.

Accordingly, in Torre de Pedra, a county with two VACV zoonotic outbreaks registered in 2007 and 2010 [3,6], no viral DNA were detected in blood samples, neither in feces or urine samples of wild rodents (Peres et al., unpublished data). On the other hand, this county showed the greatest proportion of seropositivity for cows (39%) and horses (22%), while 10% opossum were seropositive and all wild rodents were seronegative [18]. Comparatively, Bofete showed detection of viral DNA in blood samples of one cow and two *D. aurita*, and in feces of two *O. flavescens* and one *O. nigripes* (unpublished work [26]). Bofete also showed the second highest seropositive rate of cows (14%) and horses (9%), 9% of opossums (*D. albiventris* and *D. aurita*) seropositive, and the highest seropositive rate in wild rodents (11%) [18]. In contrast, in Anhembi, VACV DNA in blood samples were detected in three cows, one horse and one *D. albiventris,* while viral DNA were detected in feces samples from one *O. nigripes* and two *S. angouya* and in urine samples from one *O. flavescens* (unpublished work [26]). In addition, Anhembi showed the lowest seropositive rate in cows (5%) and horses (3%), and opossums (6.5%) and the second highest rate in wild rodents, with 9% of seropositivity [18].

When considering our previous results of serology associated with the outbreak situation, counties localization, and viral DNA detection, we can observe that Torre de Pedra, which had a VACV outbreak, showed a high prevalence of neutralizing antibodies in domestic animals, followed by Bofete and Anhembi (lower percentage of seropositivity, but VACV DNA animals positive in both counties). This is compatible with the model of endemic infection knows as the SIR model [27], where after the outbreaks in Torre de Pedra, the number of removals (R) increased due to long-term immunity promoted by VACV, resulting in reduction of susceptibles (S) and infectives (I), justifying viral DNA absence in sampled species. In contrast, the findings of Bofete and Anhembi suggests that there was still a large number of susceptibles (S = DNA negative and seronegative), which were acquired the infection (I = DNA positive), thus resulting in a progressive increase of removals (R = seropositive). In addition, this suggests the existence of a viral spread by contiguity, from Torre de Pedra to Bofete to Anhembi. It is possible that this viral circulation occurs in opossums and wild rodents, and these last animals eliminate low viral loads in their feces and urine (unpublished work [26]) contaminating the environment. The low viral loads in the environment can induce subclinical infections, which is evidenced by presence of viral DNA and neutralizing antibodies in blood of domestic and wild animals that are apparently healthy. Despite wild rodents eliminating the DNA virus in their feces, this was not evidenced in Torre de Pedra, probably due to small sample size in this county, or the average life span of these wild rodent species [28], also evidenced by the absence of positivity in both the PCR testing of blood and the Plaque Reduction Neutralizing Test (PRNT) in this county, suggesting that the virus is no longer circulating in this county.

Spread of infectious agents by contiguity is based on having a shred edge [29]. In these sense, Torre de Pedra, Bofete and Anhembi are neighboring municipalities (Figure 1). Most of the emerging infections usually originate in one geographic location and then disseminate to new places [30], so the origin could be the Torre de Pedra outbreaks. A factor that has been attributed to the spread of the VACV to other regions is the movement of bovine cattle, i.e., the buying and selling of animals between neighboring municipalities, even among states [25,31,32]. Then, the movement of herds among municipalities can promote viral spread. In addition, if the reservoir host becomes more widely disseminated, the infectious agent can appear in new places [30]. Accordingly, both opossums (*D. albiventris* and *D. aurita*) and wild rodents (*O. nigripes, O. flavescens* and *S. agouya*) were the most sampled wild mammals (Table 2). The factors involved in the interaction between them are still unclear, but may include contact with feces, social interactions, and predation, among others, as previously suggested in the murine transmission model proposed by Abrahão et al. [13].

The possible role of rodents and opossums in the spread and environmental maintenance of VACV has been suggested [13,16,19], as well as the transmission of subclinical infection between sentinel mice exposed to feces of experimentally infected mice [15,17], which reinforces our suggestion of subclinical viral spread through the feces and urine of wild rodents.

In attempt to confirm our suggestion that the virus of Torre de Pedra outbreak is the same that is circulating as a subclinical infection in wild and domestic animals from the studied region, all blood samples that were positive in amplification of the *vgf* gene were submitted to the amplification of the gene that encodes viral hemagglutinin (*ha*) [33]. According to the amino acid deletion in the *ha* gene, it is possible to classify the Brazilian VACV into two groups [11,12], which allow us to infer the origin of our virus. All tested samples results were negative to amplification of *ha* gene, and we attribute this to the possible low viral load circulating in this animals as well as the vgf targeting PCR would be more sensitive than HA targeting PCR once vgf is duplicated at most of VACV genomes, which could improve its detection by PCR. The ha protocol is less sensitive and does not detect low viral loads, which reinforces our hypothesis of low viral loads circulating in animals with subclinical infections. As mentioned in materials and methods, all samples that resulted positive in *vgf* gene amplification were submitted to cloning because the DNA concentration was insufficient to allow the direct sequencing. This low DNA concentration in the *vgf* PCR products can be possible due to low viral load in the blood of subclinical infected animals.

In attempt to increase the viral load, and although not mentioned in materials and methods, all positive samples were submitted to virus isolation in Vero cell culture at Biologic Institute of São Paulo State, which would allow amplification and sequencing of *ha* gene. After six passages in Vero cell cultures, no cytopathic effect was observed in any inoculated sample. We justify this to the low viral load in the blood of apparently health animals, evidenced by the low DNA concentrations observed in PCR tests.

Accordingly, the presence of viral DNA in blood samples of apparently healthy animals, does not mean that viable viral particles are present in the blood. Previous reports demonstrated that persons vaccinated against smallpox with the Dryvax vaccin, had VACV DNA detected in the blood, while the infectious virus were not detected in viral isolation [21]. The authors also referred that the isolation of VACV in blood samples of vaccine recipients was only observed in persons with moderate or severe complications of Vaccinia vaccine [21]. Then it is possible that viable viral particles are not present in the blood during subclinical infections.

The second possibility is that viable viral particles are not detected in the blood with the same frequency at which the viral DNA is detected. Rivetti Jr et al. [22] showed that one from eight experimentally infected cows were positive in VACV isolation in cell culture only on the fourth day post-inoculation, during 70 days of blood samples collection and analysis while an intermittent detection of VACV DNA was observed between the 2nd and 15th days post-inoculation. The absence of viable viral particles and the low DNA detection in sampled blood samples could justify both of these results.

Despite our efforts to infer about the source of the VACV found in blood samples of domestic and wild mammals in the present study, it was not possible infer any similarity to the VACV previously detected in Torre de Pedra. However, the results of *vgf* gene analysis allowed us to characterize the virus detected in blood samples of domestic and wild mammals as a Vaccinia virus. Our results corroborate previous findings that the VACV induces systemic infection with viral DNA detection in the blood [22], as well as subclinical infection in experimentally and naturally infected animals [15,17,19], and suggest that animals in the present study experience subclinical infection. Additionally, these data suggest that the virus may be circulating by contiguity in this region, but more studies are needed to confirm this.

## Figures and Tables

**Table 1:** Total of collected blood samples and PCR positivity among species.

Species	*n*	*p*	(%)
Human	148	0	(0,0)
Cow	688	4	(0,6)
Sheep	44	0	(0,0)
Swine	22	0	(0,0)
Horse	117	1	(0,8)
Dog	114	0	(0,0)
Cat	7	0	(0,0)
*Didelphis albiventris*	57	1	(1,7)
*Didelphis aurita*	16	2	(12,5)
*Gracilinanus microtarsus*	6	0	(0,0)
*Cerdocyon thous*	4	0	(0,0)
*Nasua nasua*	4	0	(0,0)
*Leopardus pardalis*	1	0	(0,0)
*Akodon montensis*	4	0	(0,0)
*Nectomys squamipes*	4	0	(0,0)
*Calomys tener*	4	0	(0,0)
*Sooretamys angouya*	13	0	(0,0)
*Oligoryzomys flavescens*	17	0	(0,0)
*Oligoryzomys nigripes*	61	0	(0,0)
TOTAL	1331	8	(0,6)

*n* = collected samples; *p* = positive samples; (%) = percentage of positives.

**Table 2:** Blood samples distribution among municipalities and proportion of PCR positives between different species.

Species	Anhembi		Bofete		Torre de Pedra	
	*n*	*p* (%)	*n*	*p* (%)	*n*	*p* (%)
Human	82	0 (0,0)	38	0 (0,0)	28	0 (0,0)
Cow	332	3 (0,9)	204	1 (0,5)	152	0 (0,0)
Sheep	33	0 (0,0)	9	0 (0,0)	2	0 (0,0)
Swine	9	0 (0,0)	12	0 (0,0)	1	0 (0,0)
Horse	71	1 (1,4)	23	0 (0,0)	23	0 (0,0)
Dog	56	0 (0,0)	35	0 (0,0)	23	0 (0,0)
Cat	3	0 (0,0)	2	0 (0,0)	2	0 (0,0)
*D. albiventris*	31	1 (3,2)	19	0 (0,0)	7	0 (0,0)
*D. aurita*	0	0 (0,0)	13	2 (15,4)	3	0 (0,0)
*G. microtarsus*	2	0 (0,0)	2	0 (0,0)	2	0 (0,0)
*C. thous*	3	0 (0,0)	1	0 (0,0)	0	0 (0,0)
*N. nasua*	3	0 (0,0)	1	0 (0,0)	0	0 (0,0)
*L. pardalis*	1	0 (0,0)	0	0 (0,0)	0	0 (0,0)
*A. montensis*	0	0 (0,0)	4	0 (0,0)	0	0 (0,0)
*N. squamipes*	3	0 (0,0)	1	0 (0,0)	0	0 (0,0)
*C. tener*	0	0 (0,0)	2	0 (0,0)	2	0 (0,0)
*S. angouya*	13	0 (0,0)	0	0 (0,0)	0	0 (0,0)
*O. flavescens*	11	0 (0,0)	3	0 (0,0)	3	0 (0,0)
*O. nigripes*	30	0 (0,0)	26	0 (0,0)	5	0 (0,0)
TOTAL	683	5 (0,7)	395	3 (0,7)	253	0 (0,0)

*n* = collected sampled; *p* = positive samples; (%) = percentage of positive samples.

**Table 3:** Correlation between Vaccinia virus (VACV) DNA detection in blood samples by PCR test and detection of neutralizing antibodies in serum samples of the same sampled animals previously tested [18].

Serological Condition	PCR Positive (%)	PCR Negative (%)	Total (%)
Seropositive ^1^	1 * (0,5)	185 (99,5)	186 (100)
Seronegative ^1^	7 ** (0,6)	1138 (99,4)	1145 (100)
Total	8 (0,6)	1323 (99,4)	1331 (100)

* Cow both positive, PCR and serology (OPV neutralizing antibodies titer equal to 16); ** PCR positive but seronegative animals (three cows, one horse, one *D. albiventris*, two *D. aurita*); **^1^** Data from previous serologic study [18].

**Table 4:** Farms in which VACV DNA were detected by PCR in blood samples and previous seropositivity of animals and humans from these DNA positive farms.

Milking Farms		PCR Positive Animals	Previous Seropositive ^1^		
			Species	*p* (%)	*n*
Anhembi	A16	Opossum (*D. albiventris*)	Human	1 (25)	4
			Domestic dog	1 (50)	2
	A2	Cow	Cow	1 (5)	20
	A23	Cow	Cow	1 (6,6)	15
		Horse			
	A7	Cow	Human	1 (16,6)	6
			Cow	1 (5)	20
			Domestic dog	2 (33,3)	6
			Wild rodent (*O. nigripes*)	1 (5,5)	18
Bofete	B46	Cow *	Horse	1 (25)	4
			Domestic dog	1 (16,6)	6
			Cows	5 (25)	20
	B47	Opossum (two *D. aurita*)	Human	1 (50)	2
			Swine	2 (33,3)	6

* Cow positive in both tests, PCR and Serology; **^1^** Data from previous serologic study [18], *p* = number of previous seropositive; *n* = sampled individuals from each species; (%) = percentage of seropositives.

## Supplementary Materials

Supplementary materials can be found at www.mdpi.com/1999-4915/10/1/42/s1.
