# Supplementary material for: Vaccinia Virus in Blood Samples of Humans, Domestic and Wild Mammals in Brazil
Source: Viruses. 2018 Jan 18;10(1):42. doi: 10.3390/v10010042 (PMC5795455; doi:10.3390/v10010042)
Supplement: Supplementary file 1 [file viruses-10-00042-s001.pdf]

Table S1. Percentage of identity between the Vaccinia virus (VACV) DNA positive samples from different milking farms

	225	270	263	693	706	211
270	97%					
263	96%	96%				
693	96%	97%	96%			
706	96%	97%	96%	97%		
211	96%	96%	95%	96%	96%	
694	96%	97%	97%	96%	97%	96%
